# Nonrandom divergence of gene expression following gene and genome duplications in the flowering plant *Arabidopsis thaliana*

**DOI:** 10.1186/gb-2006-7-2-r13

**Published:** 2006-02-20

**Authors:** Tineke Casneuf, Stefanie De Bodt, Jeroen Raes, Steven Maere, Yves Van de Peer

**Affiliations:** 1 Department of Plant Systems Biology, Flanders Interuniversity Institute for Biotechnology (VIB), Ghent University, Technologiepark 927, B-9052 Ghent, Belgium; 2 Computational and Structural Biology Unit, European Molecular Biology Laboratory (EMBL), Meyerhofstrasse, D-69117 Heidelberg, Germany

## Abstract

**Background:**

Genome analyses have revealed that gene duplication in plants is rampant. Furthermore, many of the duplicated genes seem to have been created through ancient genome-wide duplication events. Recently, we have shown that gene loss is strikingly different for large- and small-scale duplication events and highly biased towards the functional class to which a gene belongs. Here, we study the expression divergence of genes that were created during large- and small-scale gene duplication events by means of microarray data and investigate both the influence of the origin (mode of duplication) and the function of the duplicated genes on expression divergence.

**Results:**

Duplicates that have been created by large-scale duplication events and that can still be found in duplicated segments have expression patterns that are more correlated than those that were created by small-scale duplications or those that no longer lie in duplicated segments. Moreover, the former tend to have highly redundant or overlapping expression patterns and are mostly expressed in the same tissues, while the latter show asymmetric divergence. In addition, a strong bias in divergence of gene expression was observed towards gene function and the biological process genes are involved in.

**Conclusion:**

By using microarray expression data for *Arabidopsis thaliana*, we show that the mode of duplication, the function of the genes involved, and the time since duplication play important roles in the divergence of gene expression and, therefore, in the functional divergence of genes after duplication.

## Abstract 2

Analysis of expression data of duplicated genes in *Arabidopsis thaliana *shows that the mode of duplication, the time since duplication and the function of the duplicated genes play a role in the divergence of their expression.

## Background

Recent studies have revealed a surprisingly large number of duplicated genes in eukaryotic genomes [1,2]. Many of these duplicated genes seem to have been created in large-scale, or even genome-wide duplication events [3,4]. Whole genome duplication is particularly prominent in plants and most of the angiosperms are believed to be ancient polyploids, including a large proportion of our most important crops such as wheat, maize, soybean, cabbage, oat, sugar cane, alfalfa, potato, coffee, cotton and tobacco [5-8]. For over 100 years, gene and genome duplications have been linked to the origin of evolutionary novelties, because it provides a source of genetic material on which evolution can work ([9] and references therein). In general, four possible fates are usually acknowledged for duplicated genes. The most likely fate is gene loss or nonfunctionalization [1,10-12], while in rare cases one of the two duplicates acquires a new function (neofunctionalization) [13]. Subfunctionalization, in which both gene copies lose a complementary set of regulatory elements and thereby divide the ancestral gene's original functions, forms a third potential fate [14-17]. Finally, retention is recognized for two gene copies that, instead of diverging in function, remain largely redundant and provide the organism with increased genetic robustness against harmful mutations [18-20].

The functional divergence of duplicated genes has been extensively studied at the sequence level to investigate whether genes evolve at faster rates after duplication, or are under positive or purifying selection [21-26]. The recent availability of functional genomics data, such as expression data from whole-genome microarrays, opens up completely novel ways to investigate the divergence of duplicated genes, and several studies using such data have already provided intriguing new insights into gene fate after duplication. In yeast, for instance, Gu and co-workers [27] found a significant correlation between the rate of coding sequence evolution and divergence of expression and showed that most duplicated genes in this organism quickly diverge in their expression patterns. In addition, they showed that expression divergence increases with evolutionary time. Makova and Li [28] analyzed spatial expression patterns of human duplicates and came to the same conclusions. They calculated the proportion of gene pairs with diverged expression in different tissues, and found evidence for an approximately linear relationship with sequence divergence. Wagner [29] showed that the functional divergence of duplicated genes is often asymmetrical because one duplicate frequently shows significantly more molecular or genetic interactions/functions than the other. Adams and co-workers [30] examined the expression of 40 gene pairs duplicated by polyploidy in natural and synthetic tetraploid cotton and showed that, although many pairs contributed equally to the transcriptome, a high percentage exhibited reciprocal silencing and biased expression and were developmentally regulated. In a few cases, genes duplicated through polyploidy events were reciprocally silenced in different organs, suggesting subfunctionalization.

In *Arabidopsis*, Blanc and Wolfe [31] investigated the expression patterns of genes that arose through gene duplication and found that about 62% of the recent duplicates acquired divergent expression patterns, which is in agreement with previous observations in yeast and human. In addition, they identified several cases of so-called 'concerted divergence', where single members of different duplicated genes diverge in a correlated way, resulting in parallel networks that are expressed in different cell types, developmental stages or environmental conditions. Also in *Arabidopsis*, Haberer *et al*. [32] studied the divergence of genes that originated through tandem and segmental duplications by using massively parallel signature sequencing (MPSS) data and concluded that, besides a significant portion of segmentally and tandemly duplicated genes with similar expression, the expression of more than two-thirds of the duplicated genes diverged in expression. However, expression divergence and divergence time were not significantly correlated, as opposed to findings in human and yeast (see above). In a small-scale study on regulatory genes in *Arabidopsis*, Duarte *et al*. [33] performed an analysis of variance (ANOVA) and showed that 85% of the 280 paralogs exhibit a significant gene by organ interaction effect, indicative of sub- and/or neofunctionalization. Ancestral expression patterns inferred across a type II MADS box gene phylogeny indicated several cases of regulatory neofunctionalization and organ-specific nonfunctionalization.

In conclusion, recent findings demonstrate that a majority of duplicated genes acquire different expression patterns shortly after duplication. However, whether the fate of a duplicated gene also depends on its function is far less understood. The model plant *Arabidopsis *has a well-annotated genome and, in addition to many small-scale duplication events, there is compelling evidence for three genome duplications in its evolutionary past [34-37], hereafter referred to as 1R, 2R, and 3R. Recently, a nonrandom process of gene loss subsequent to these different polyploidy events has been postulated [12,31,38]. Maere *et al*. [12] have shown that gene decay rates following duplication differ considerably between different functional classes of genes, indicating that the fate of a duplicated gene largely depends on its function. Here, we study the expression divergence of genes that were created during both large- and small-scale gene duplication events by means of two compiled microarray datasets. The influence of the origin (mode of duplication) and the function of the duplicated genes on expression divergence are investigated.

## Results and discussion

**Figure 1:**
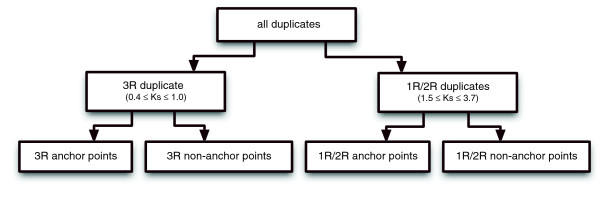
The duplicated genes of *Arabidopsis thaliana *were divided into six different subclasses according to the time and mode of duplication (see Materials and methods for details).

To examine general gene expression divergence patterns, we analyzed two datasets containing genome-wide microarray data for *Arabidopsis *genes (see Materials and methods). The first consisted of 153 Affymetrix ATH1 slides with expression data of various perturbation and knockout experiments (see Additional data file 1). The Spearman rank correlation coefficient was computed between the two expression patterns of every duplicated gene pair. To investigate whether divergence of gene expression varies for duplicates that were created by small-scale or large-scale (genome-wide) events, the complete set of duplicated genes was subdivided into different subgroups and their expression correlation was examined (see Materials and methods; Figure 1). We refer to anchor genes as duplicated genes that are still lying in recognizable duplicated segments. Such anchor-point genes, and consequently the segments in which they reside, are regarded as being created in large-scale duplication events. Six different sets of genes were distinguished: one set containing duplicates with ages corresponding to 1R/2R (1.5 ≤ Ks ≤ 3.7), further subdivided into two sets of anchor and non-anchor points, and one set of younger duplicates with ages corresponding to 3R (0.4 ≤ Ks ≤ 1.0), again subdivided into two sets of anchor and non-anchor points (see Materials and methods). Differences in expression divergence between anchor points and non-anchor points were evaluated by comparing their distributions of correlation coefficients using a Mann Whitney *U* test (see Materials and methods). We further explored the difference between both classes of genes by means of a second dataset on tissue-specific expression (see Materials and methods and Additional data file 2) [39]. Here, for each of the subgroups of duplicates described above we calculated present/absent calls in the 63 different tissues and computed both the absolute and relative amount of tissues in which the two genes of a duplicated gene pair are expressed.

In addition, the first dataset was used to identify possible biases toward gene function. The expression correlation of duplicated gene pairs, represented by the Spearman correlation coefficient, was studied in relation to the age of duplication, represented by K_S _(amount of synonymous substitutions per synonymous site) for genes belonging to different functional categories (GO slim, see Materials and methods).

### Divergence of expression and mode of duplication

**Figure 2:**
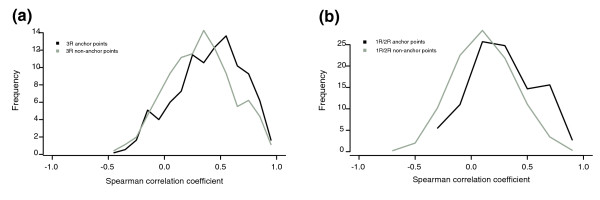
Histograms of the Spearman correlation coefficients for anchor points (black) and non-anchor points (grey) for both **(a) **3R genes and **(b) **1R/2R genes. A Mann-Whitney *U* test was used to test whether both distributions are significantly different from each other. Mean correlation coefficients: 0.40 for 3R anchor points; 0.32 for 3R non-anchor points; 0.28 for 1R/2R anchor points; and 0.11 for 1R/2R non-anchor points.

**Figure 3:**
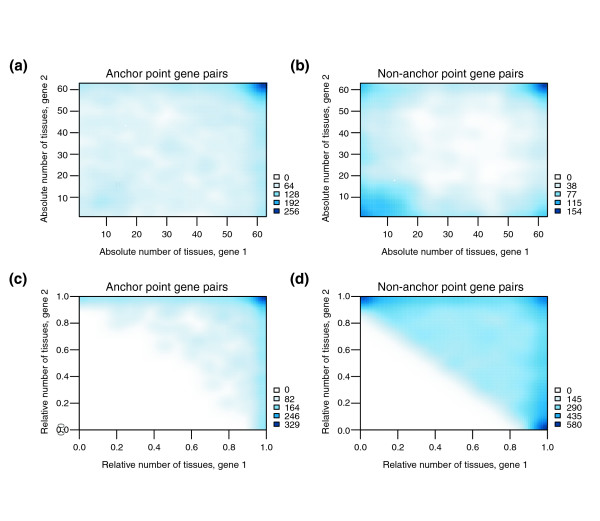
Smoothed color density representations of the scatterplots of the **(a,b) **absolute and **(c,d) **relative numbers of tissues in which the genes of a duplicated gene pair are expressed, for both (a,c) 3R anchor points and (b,d) non-anchor points. From (a,c) we can conclude that many anchor point genes are both expressed in a high number of tissues, and that many of these tissues are actually identical. On the other hand, (b,d) show that non-anchor point genes frequently show asymmetric divergence because many genes are expressed in a high number of tissues, while their duplicate is not. The plots were made using the 'smoothScatter' function, implemented in the R package 'prada' [69], by binning the data (in 100 bins) in both directions. The intensity of blue represents the amount of points in the bin, as depicted in the legend.

**Figure 4:**
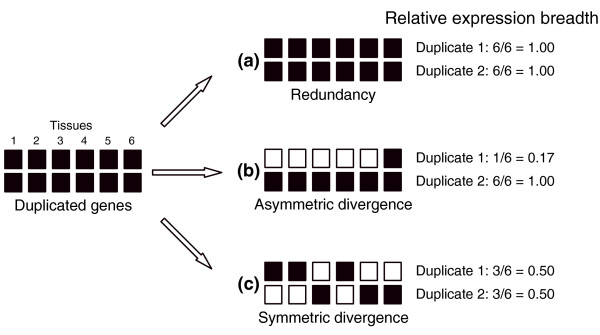
Hypothetical example showing possible scenarios for tissue-specific expression of two duplicates. A black box depicts expression in a particular tissue, whereas a white box represents no expression in that particular tissue. Following duplication of a gene that is expressed in six different tissues, the two copies can **(a) **both remain expressed in all six tissues (redundancy), **(b) **diverge asymmetrically, where one gene is expressed in only a small subset of the tissues, while its duplicate remains expressed in the original six tissues, or **(c) **diverge symmetrically, where tissue-specific expression is complementarily lost between both duplicates. The absolute number of tissues in which a gene is expressed is six for both duplicates in (a) and for the second duplicate in (b), one for the first duplicate in (b) and three for both duplicates in (c). The total number of tissues in which the pair is expressed is 6 in all three cases. The relative number is the fraction of the previous two, and is 1 for the two genes in (a) and for the second duplicate in (b), 0.17 for the first duplicate in (b) and 0.5 for both duplicates in (c).

First, we investigated whether the mode of duplication that gives rise to the duplicate gene pairs affects expression divergence. Interestingly, for both younger (Figure 2a) and older (Figure 2b) duplicates, anchor points showed a significantly higher correlation in expression than non-anchor points (*p* values of 2.49e^-07 ^and 1.67e^-08 ^for young and old genes, respectively). Even for the younger duplicates the difference is striking (Figure 2a). We explored the second dataset on tissue-specific expression and first considered the absolute number of tissues in which genes are expressed, resembling the expression breadth (see Materials and methods). Regarding anchor points, both genes are usually expressed in a high number of tissues (Figure 3a). This is only partly true for non-anchor points (or genes assumed to have been created in small-scale duplications), where many duplicates are expressed in a much smaller number of tissues (shown for young duplicates in Figure 3b). To further discriminate between redundancy, complementarity and asymmetric divergence, and thus to investigate if genes are expressed in the same tissues, we computed the relative number of tissues a gene is expressed in, which is the number of tissues in which a gene is expressed divided by the total number of tissues in which either one of the two duplicates is expressed. As schematically represented in Figure 4, two duplicated genes that remain co-expressed in the same tissues will both have a relative number equal to 1 (redundant genes; Figure 4a), whereas asymmetrically diverged genes, where one gene is expressed in a very small number of tissues as opposed to its duplicate that is expressed in a high number of tissues, can be identified by relative numbers close to 0 and close to 1, respectively (Figure 4b). The intermediate situation, where two duplicate genes are expressed in an equal number of different tissues, will result in both copies having a relative number equal to 0.5 (Figure 4c). When assuming that the ancestral gene was expressed in all tissues in which the two duplicate genes are expressed, the latter case hints at subfunctionalization after duplication. Figure 3c,d shows these relative numbers for 3R anchor points and non-anchor points, respectively, and show that redundancy is much more common among anchor points (Figure 3c) than among non-anchor points (Figure 3d) of similar ages. Moreover, gene pairs resulting from small-scale duplications not only seem to have diverged more often than those created by segmental or genome duplications, but they also have diverged asymmetrically, where one gene is expressed in a high number of tissues, as opposed to its duplicate that is expressed in a small number of tissues (Figure 3d, top left and bottom right). Similar findings on tissue-specific expression were observed for the 1R/2R genes (results not shown).

The current study clearly shows that duplicated genes that are part of still recognizable duplicated segments (so-called anchor points) show higher correlation in gene expression than duplicates that do not lie in paralogons, despite their similar ages. In addition, the former have highly redundant or overlapping expression patterns, as they are mostly expressed in the same tissues. This is in contrast with what is observed for the non-anchor point genes, where asymmetric divergence is more widespread. There might be several explanations for these observations. The set of non-anchor point genes include genes created by tandem duplication, transpositional duplication, or genes translocated after segmental duplication events. One explanation might lie in different gene duplication mechanisms. Single-gene duplications, mostly caused by unequal crossing-over and duplicative transposition [40], are much more prone to promoter disruption than genes duplicated through polyploidy events, which might lead to the altered (or observed asymmetric) expression of genes after small-scale gene duplication events. Similarly, translocation of genes that originated from large-scale duplication events can also disrupt promoters, again contributing to the overall increase of expression divergence [41,42].

Alternatively, the higher correlation of anchor points might result directly from co-expression of neighboring genes, regardless of their involvement in the same pathway, as shown recently by Williams and Bowles [43]. It was also shown that genome organization, and more in particular the chromatin structure, can affect gene expression [43-48]. Such additional structural and functional constraints might, therefore, reduce the freedom to diverge and, as a consequence, cause the expression patterns of genes in duplicated regions to remain similar, as observed here. Related to our observations, Rodin *et al*. ([49] and references therein) reported that position effects play an important role in the evolution of gene duplicates. Repositioning of a duplicate to an ectopic site is proposed to epigenetically modify its expression pattern, along with the rate and direction of mutations. This repositioning is believed to rescue redundant anchor point genes from pseudogenization and accelerate their evolution towards new developmental stage-, time-, and tissue-specific expression patterns [49].

As previously stated, non-anchor point genes not only appear to show higher expression divergence than anchor-point genes, they appear to diverge asymmetrically, where one gene is expressed in a high number of tissues, while its duplicate is expressed in a lower number of tissues. It should be noted that we cannot establish whether one duplicate is becoming highly specialized and dedicated to a very small number of tissues or whether it is losing much of its functionality (that is, turning into a pseudogene), nor can we distinguish between the gain of expression in new tissues for one gene versus the loss of expression for the other gene duplicate, as we would therefore need to know the expression pattern of the ancestral gene. In this respect, it is interesting to note that it is currently not known whether the ancient genome doublings in (the ancestor of) *A. thaliana *resulted from auto- or allopolyploidization. In the former case, the anchor point duplicates are in fact real paralogs, while in the latter case the expression of the two gene copies might have (slightly) differed from the start ([50,51] and references therein). Nevertheless, our data clearly show that the duplicates that still lie in duplicated segments show high expression correlation and have highly overlapping expression patterns, as opposed to those that arose through small-scale duplication events or have been translocated afterwards.

In concordance with the results discussed above, Wagner [29] described asymmetric divergence of duplicated genes in the unicellular organism *Saccharomyces cerivisiae*. He reported that both the number of stressors to which two duplicates respond and the number of genes that are affected by the knockout of paralogous genes are asymmetric. He therefore proposed an evolutionary model in which the probability that a loss-of-function mutation has a deleterious effect is greatest if the two duplicates have diverged symmetrically. Asymmetric divergence of genes therefore leads to increased robustness against deleterious mutations. This seems to be confirmed by our results. Indeed, also in *A. thaliana*, asymmetric divergence, rather than symmetric divergence, seems to be the fate for two duplicates, at least when they do not lie in duplicated segments.

### Divergence of expression and gene function

**Figure 5:**
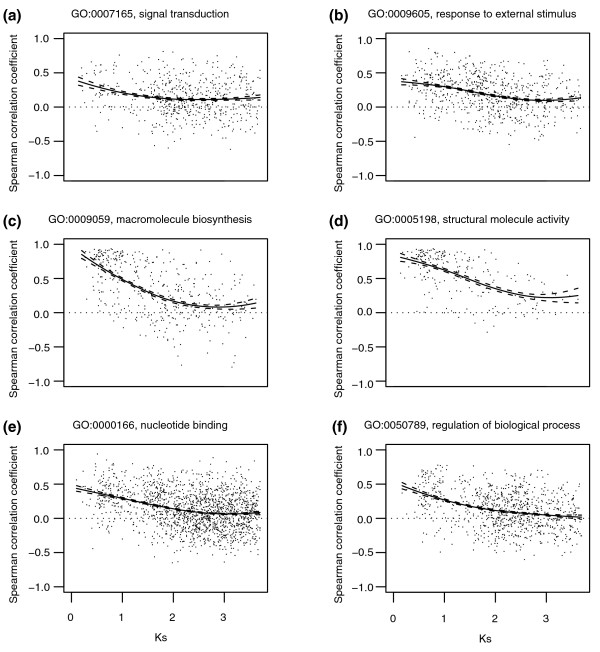
Scatter plots of the correlation coefficient in function of the K_S _value of the gene pairs belonging to different functional classes. The full black line represents the local regression (locfit) line fitted to the data of that particular class, together with its 95% confidence interval (dashed line). **(a-b) **Gene pairs that have diverged quickly after birth have an intercept of the regression line with the y-axis close to zero; **(c-d) **whereas slow divergence is reflected by an intercept with the y-axis close to one and a steep slope. **(e-f) **A more average situation can be observed for most classes. Data of the following classes are displayed: (a) signal transduction; (b) response to external stimuli; (c) macromolecule biosynthesis; (d) structural molecule activity; (e) nucleotide binding; (f) regulation of biological process. Plots of other functional classes of genes can be found in Additional data file 3.

Next, we studied how the expression correlation, measured as the Spearman correlation coefficient, changes over time for genes of ages up to a K_S _of 3.7. Loess smoothers, which locally summarize the trend between two variables (see full black lines in Figure 5), clearly indicate that correlation of expression, in general, is high for recently duplicated genes, declines as time increases, and saturates at a certain time point. Interestingly, considerable differences can be observed between genes belonging to different functional classes (Figure 5; Additional data file 3). For example, genes that are involved in signal transduction and response to external stimulus appear to have diverged very quickly after duplication (Figure 5a,b, respectively). Similar trends can be observed for genes involved in response to biotic stimuli and stress, cell communication, carbohydrate and lipid metabolism, and for genes with hydrolase activity (Additional data file 3). Interestingly, genes of many of these classes are involved in reactions against environmental changes or stress (signal transduction, cell communication, response to external and biotic stimuli and stress, lipid metabolism), which might suggest that *Arabidopsis *(or better its ancestors) quickly put these newborn genes into use by means of altered and diverged expression patterns, as compared to their ancestral copy, to survive and cope with environmental changes.

Slowly diverging expression patterns were found for proteins involved in, for example, macromolecule biosynthesis (Figure 5c) and structural molecule activity (Figure 5d) as reflected in the large number of young gene pairs with high correlation coefficients. Analogous trends can be observed for other functional classes containing genes involved in cell organization and biogenesis, nucleic acid, macromolecule, protein and primary metabolism, biosynthesis and response to endogenous stimulus (Additional data file 3). Apparently, although duplicated genes within these classes are being retained, their fast diversification at the expression level is selected against, probably due to the essential nature and sensitive regulation of these highly conserved processes. Other classes of genes, like those having nucleotide binding capacity (Figure 5e) and those involved in regulation of biological processes (Figure 5f), show moderate divergence rates. The DNA binding, transcription, protein modification, and genes with catalytic, transcription factor and transporter activity (Additional data file 3) classes of genes show similar divergence patterns. We also tested whether the divergence patterns described above are significantly different from each other by interchanging the fitted models between functional classes (fit the locfit line of a particular class to the data of another class) and evaluating the model quality. Our results confirmed that there are indeed significant differences between slowly, moderately and quickly diverging genes (results not shown).

As opposed to Haberer *et al*. [32], but in agreement with Gu *et al*. [27] and Makova and Li [28], who described expression divergence of duplicated genes in yeast and human, respectively, we here show that in *Arabidopsis*, expression patterns of duplicates diverge as time increases. In addition, the rate of divergence seems to be highly dependent on the molecular function of the gene or the biological process in which it is involved. The rate of expression divergence ranges from very slow, for highly conserved proteins, such as ribosomal proteins, or genes involved in conserved processes, such as biosynthesis pathways or photosynthesis, to very quickly, for instance genes involved in adaptation to and reaction against changing environments.

Note that, because we removed expression data of genes without a unique probeset (see Materials and methods), there are actually more young duplicates than the ones that were plotted in Figure 5. Although the current microarray technology does not allow measuring their expression, we can assume that their presence would increase the overall correlation, especially in the low value range of K_S_. As the difficulty to design a gene-specific probeset is not related to the functional class, we assume that all functional classes suffer from this caveat to the same extent and that the differences we observe are reliable.

## Conclusion

Investigating gene and genome duplication events as well as the subsequent functional divergence of genes is of fundamental importance in the understanding of evolution and adaptation of organisms. Previously, large-scale gene duplication events have been shown to be prominent in different plant species. Only recently, a pattern of gene retention after duplication has emerged that is biased towards function, time and mode of duplication [5,12,38]. For instance, genes involved in signal transduction and transcriptional regulation were shown to have been preferentially retained after large-scale duplication events, while genes of other important functional categories (such as DNA metabolism and cell cycle) were lost [5,12,38]. Still other categories of genes, such as those involved in secondary metabolism, are highly retained after small-scale gene duplication [12]. Here, we have studied the expression divergence of these retained duplicates by means of the genome-wide microarray expression data available for *Arabidopsis *genes. As clearly shown in the current study, there is not only a bias in the retention of genes after duplication events, but also in the rate of divergence of expression for different functional categories of genes. Surprisingly, this bias is much more outspoken for genes created by small-scale duplication events than for genes that have been created through large-scale segmental or entire genome duplication events. The latter genes, provided they are still found in duplicated segments, show much higher expression correlation and highly overlapping expression patterns compared to those duplicates that are created by small-scale duplication events or that no longer lie in duplicated segments.

## Materials and methods

### Duplicated genes

To identify duplicated genes, an all-against-all protein sequence similarity search was performed using BLASTP (with an E-value cut-off of e^-10^) [52]. Sequences alignable over a length of 150 amino acids with an identity score of 30% or more were defined as paralogs according to Li *et al*. [53]. To determine the time since duplication, the fraction of synonymous substitutions per synonymous site (K_s_) was estimated. These substitutions do not result in amino acid replacements and are, in general, not under selection. Consequently, the rate of fixation of these substitutions is expected to be relatively constant in different protein coding genes and, therefore, to reflect the overall mutation rate. First, all pairwise alignments of the paralogous nucleotide sequences belonging to a gene family were made by using CLUSTALW [54], with the corresponding protein sequences as alignment guides. Gaps and adjacent divergent positions in the alignments were subsequently removed. K_S _estimates were then obtained with the CODEML program [55] of the PAML package [56]. Codon frequencies were calculated from the average nucleotide frequencies at the three codon positions (F3 × 4), whereas a constant K_N_/K_S _(nonsynonymous substitutions per nonsynonymous site over synonymous substitutions per synonymous site, reflecting selection pressure) was assumed (codon model 0) for every pairwise comparison. Calculations were repeated five times to avoid incorrect K_S _estimations because of suboptimal local maxima.

To compare expression patterns of duplicated genes that had arisen through genome duplication events with those created in small-scale duplication events, the complete set of duplicated genes was subdivided into six different subgroups (Figure 1), namely:

1. Set 1 containing all genes that are assumed to have been duplicated at a time coinciding with the most recent (3R) polyploidy event.

2. Set 2 containing all genes that are assumed to have been duplicated at a time coinciding with the two (1R/2R) older polyploidy events.

3. Set 3 is a subset of Set 1 and only contains the anchor points (pairs of duplicated genes that still lie on so-called paralogons [34], homologous duplicated segments that still show conserved gene order and content). These genes are thus assumed to have been created by 3R.

4. Set 4 containing the non-anchor point duplicates of Set 1.

5. Set 5 containing the anchor points of Set 2 assumed to have been created by 1R/2R.

6. Set 6 containing the non-anchor points of Set 2.

Previously, through modeling the age distribution of duplicated genes, we estimated that genes created during the youngest genome duplication have a K_S _between 0.4 and 1.0, while genes that originated during the oldest two genome duplications were estimated to have a K_S _between 1.5 and 3.7 [12]. The latter genes were grouped because it was difficult to unambiguously attribute them to 1R or 2R [12,35]. ere, it is assumed that anchor points dddddThe duplicated gene pairs that arose through genome duplication events (anchor points) had been identified previously (complete list available upon request) [34].

### Gene Ontology functional classes

Duplicated genes were assigned to functional categories according to the Gene Ontology (GO) annotation. The GO annotation for *A. thaliana *was downloaded from TAIR (version 24 June 2005) [57]. We studied genes belonging to the biological process (BP) and the molecular function (MF) classes of the GO tree. Rather than considering all categories from different levels in the gene ontology, we used the plant-specific GO Slim process and function ontologies [58]. In these GO Slim ontologies, categories close to the leaves of the GO hierarchy are mapped onto the more general, parental categories. A gene pair is included in a functional class only when both genes of the pair have been assigned to that particular functional class. Functional classes containing fewer than 200 pairs of duplicated genes were excluded from the analysis.

### Microarray expression data

This study was based on gene expression data generated with Affymetrix ATH1 microarrays (Affymetrix, San Diego, CA, USA) [59] during various experiments, all of which are publicly available from the Nottingham Arabidopsis Stock Centre (NASC) [60,61]. Two datasets were examined that both comprise microarrays that were replicated at least once. The first set includes 153 microarrays that were generated under a broad range of experimental conditions, including, for example, diverse knockout mutants and chemical and biological perturbations (Additional data file 1). Raw data were subjected to robust multi-array average (RMA) normalization, which is available through Bioconductor [62,63]. The probe set data of all arrays were simultaneously normalized using quantile normalization, which eliminates systematic differences between different chips [64-66]. The log-transformed values were used instead of the raw intensities because of the variance-stabilizing effect of this transformation. Because of the high sequence similarity of recently duplicated genes and the risk of artificially increased correlation due to cross-hybridization, we selected expression data only from those genes for which a unique probe set is available on the ATH1 microarray (probe sets that are designated with an '_at' extension, without suffix). Next, the genes were non-specifically filtered based on expression variability by arbitrarily selecting the 10,000 genes with the highest interquartile range. This was done in an attempt to filter out those genes that show very little variability in gene expression, thereby artificially increasing the overall expression correlation. The mean intensity value was calculated for the replicated slides, resulting in 66 data points for every gene. Next, for each of the 16 different experimental conditions, a treated plant and its corresponding wild-type plant (control experiment without treatment, knock-out or perturbation) were identified (Additional data file 1). To adjust the data for effects that arise from variation in technology rather than from biological differences between the plants, for every gene the intensity value of the wild type was subtracted from that of the treated plant. The final dataset contained 49 expression measures per gene. For each of the six subsets of duplicates described above 1,279, 8,510, 550, 708, 109, and 8,389 gene pairs, respectively, remained after filtering the microarray data.

The second dataset contains the expression data of genes in 63 plant tissues that were generated within the framework of the AtGenExpress project (Additional data file 2) [39]. The 'mas5calls' function in Bioconductor was used to study tissue-specific gene expression [62,63]. This software evaluates the abundance of each transcript and generates a 'detection *p* value', which is used to determine the detection call, indicating whether a transcript is reliably detected (present) or not (absent or marginal). The parameters used correspond to the standard Affymetrix defaults in which a gene with a *p* value of less than 0.04 is marked as 'present' [67,68]. We again selected only expression data from those genes for which a unique probe set is available on the ATH1 microarray. The dataset contains triplicated microarrays and we assigned a gene to be present if it was assigned with a present call in at least one of the three samples. In all other cases an absent call was assigned. We plotted both the absolute (or expression breadth) and relative (or expression divergence of two duplicates) number of tissues in which the genes of a duplicated gene pair are expressed. The latter is defined as the number of tissues in which a gene has a present call divided by the total number of present calls of the duplicated gene pair. Pairs of genes without any present calls were removed from the dataset, resulting in 6,193, 37,838, 1,387, 4,736, 269, 37,438 genes, respectively, for each of the six subsets described above. Both of the above described datasets are available upon request.

### Correlation analysis

To measure the expression divergence of two duplicated genes, the Spearman Rank correlation coefficient ρ was calculated. We chose to use this non-parametric statistic because our dataset is a compilation of data from uncorrelated experiments, and might therefore contain outliers. The formula used was:



where *D *is the difference between the ranks of the corresponding expression values of both duplicated genes and *N *is the number of samples. In evaluating and comparing the distributions of the correlation coefficients of the expression of a set of genes, we used the Mann-Whitney *U* test (two sided, not paired) that is incorporated in the statistical package R [69].

### Regression analysis

The relation between expression correlation, measured as the Spearman correlation coefficient, and time, measured as the number of synonymous substitutions per synonymous site K_S_, was studied using 'locfit', an R package to fit curves and surfaces to data, using local regression and likelihood methods [69,70]. We hereby included all duplicated genes with a K_S _value smaller than or equal to 3.7 (see above). A local regression model was fitted to the data of each of the functional classes of genes and we looked for biases in expression divergence between the different functional classes by interchanging the fitted models. The model fitted to the data of a particular class was fitted to the data of another class and the quality of the fit was evaluated by assessing the relation between the residuals and fitted values. Residuals that show a clear trend (which is reflected in a non-random distribution around Y = 0 with zero mean) indicate that the fitted regression model is inappropriate (that is, the model fitted to the data of the former class is not applicable to the data of the latter).

## Additional data files

The following additional data are available with the online version of this paper. Additional data file 1 is a description of dataset 1. Additional data file 2 is a description of dataset 2. Additional data file 3 presents scatterplots of genes belonging to different functional classes. Supplemental material is also available online at [71].

## Authors' contributions

TC designed the study, analyzed data, and wrote the paper. SDB analyzed data. JR designed the study. SM analyzed data. YVdP designed the study, supervised the project, and wrote the paper.

## Supplementary Material

Additional File 1 This file contains the names of the microarrays that were included in the first dataset, together with the description of the experimental conditions (that is, to what series of experiments the microarrays belong, from what type of plant the samples were taken, and to what wild type the slide should be compared). Click here for file

Additional File 2 This file contains the names of the microarrays that were included in the second dataset, together with the description of what tissue the samples were taken from and the conditions in which the plant was grown. Click here for file

Additional File 3 This file contains the scatterplots of the Spearman correlation coefficient in function of the Ks value of all genes in the 67 different functional classes of genes. The loess smoother that was fitted to the data is depicted by a full black line, together with its 95% confidence interval. Click here for file
