# Supplementary material for: Nonrandom divergence of gene expression following gene and genome duplications in the flowering plant Arabidopsis thaliana
Source: Genome Biol. 2006 Feb 20;7(2):R13. doi: 10.1186/gb-2006-7-2-r13 (PMC1431724; doi:10.1186/gb-2006-7-2-r13)
Supplement: Additional File 1 — This file contains the names of the microarrays that were included in the first dataset, together with the description of the experimental conditions (that is, to what series of experiments the microarrays belong, from what type of plant the samples were taken, and to what wild type the slide should be compared). [file gb-2006-7-2-r13-S1.pdf]

Dataset 1

Series	Slide Name	Slide type	Corresponds to
1	A1.MILL.AIR.CEL	c1	
	A1.MILL.AIR.REP2.CEL	c1	
	A2.MILL.ETH.CEL	e1	c1
	A2.MILL.ETH.REP2.CEL	e1	c1
	A3.MILL.LL.CEL	e2	c1
	A3.MILL.LL.REP2.CEL	e2	c1
	A4.MILL.AIR.REP3.CEL	c1	
	A5.MILL.ETH.REP3.CEL	e1	c1
	A6.MILL.LL.REP3.CEL	e2	c1
2	A10.Warre.Wca.CEL	e1	c1
	A11.Warre.6ca.CEL	e6	c1
	A12.Warre.6ca.CEL	e6	c1
	A13.Warre.3ca.CEL	e9	c1
	A14.Warre.3ca.CEL	e9	c1
	A15.Warre.2ca.CEL	e4	c1
	A16.Warre.2ca.CEL	e4	c1
	A17.Warre.Wdr.CEL	e2	c1
	A18.Warre.Wdr.CEL	e2	c1
	A19.Warre.6dr.CEL	e7	c1
	A1.Warre.Wna.CEL	c1	
	A20.Warre.6dr.CEL	e7	c1
	A2.Warre.Wna.CEL	c1	
	A3.Warre.6na.CEL	e5	c1
	A4.Warre.6na.CEL	e5	c1
	A5.Warre.3na.CEL	e8	c1
	A6.Warre.3na.CEL	e8	c1
	A7.Warre.2na.CEL	e3	c1
	A8.Warre.2na.CEL	e3	c1
	A9.Warre.Wca.CEL	e1	c1
3	A1.Wilson.mla.CEL	e1	c1
	A2.Wilson.mlb.CEL	e2	c2
	A3.Wilson.lea.CEL	c1	
	A4.Wilson.Ler.CEL	c2	
	A1.GVB.Rep1.CEL	e1	c1
	A1.Wilson.Rep2.CEL	e1	c1
	A2.GVB.Rep1.CEL	e2	c2
	A2.Wilson.Rep2.CEL	e2	c2
	A3.GVB.Rep1.CEL	c1	
	A3.Wilson.Rep2.CEL	c1	
	A4.GVB.Rep1.CEL	c2	
	A4.Wilson.Rep2.CEL	c2	
4	A1.WARRE.WTC.2..CEL	c1	
	A2.WARRE.WTW.CEL	c1	
	A3.WARRE.S6C.CEL	e1	c1
	A4.WARRE.S6W.2..CEL	e1	c1
	A5.WARRE.S2C.new..CEL	e2	c1
	A6.WARRE.S2W.CEL	e2	c1

5	Control.3.new.CEL	c2	
	Control.4..CEL	c2	
	Heat.3.new.CEL	e3	c2
	Heat.4..CEL	e3	c2
	Sen.3.new.CEL	e4	c2
	Sen.4..CEL	e4	c2
6	A1.1.cornah.icl.CEL	e1	c1
	A1.2.cornah.icl.CEL	e1	c1
	A1.3.cornah.icl.CEL	e1	c1
	A2.1.cornah.irv.CEL	e2	c1
	A2.2.cornah.irv.CEL	e2	c1
	A2.3.cornah.irv.CEL	e2	c1
	A3.1.cornah.msx.CEL	e3	c1
	A3.2.cornah.msx.CEL	e3	c1
	A3.3.cornah.msx.CEL	e3	c1
	A4.1.cornah.wsx.CEL	c1	
	A4.2.cornah.wsx.CEL	c1	
	A4.3.cornah.wsx.CEL	c1	
7	A1.LLOYD.POH.CEL	e1	c1
	A2.LLOYD.POH.CEL	e1	c1
	A3.LLOYD.POH.CEL	e1	c1
	A4.LLOYD.CON.CEL	c1	
	A5.LLOYD.CON.CEL	c1	
	A6.LLOYD.CON.CEL	c1	
8	A10.grevi.AT1.CEL	e3	c1
	A11.grevi.AT2.CEL	e3	c1
	A12.grevi.AT3.CEL	e3	c1
	A1.grevi.CC1.CEL	c1	
	A2.grevi.CC2.CEL	c1	
	A3.grevi.CC3.CEL	c1	
	A4.grevi.AC1.CEL	e1	c1
	A5.grevi.AC2.CEL	e1	c1
	A6.grevi.AC3.CEL	e1	c1
	A7.grevi.CT1.CEL	e2	c1
	A8.grevi.CT2.CEL	e2	c1
	A9.grevi.CT3.CEL	e2	c1
9	A1.Heggi.CAG.CEL	c1	
	A2.Heggi.CEG.CEL	e3	c1
	A3.Heggi.HAG.CEL	e2	c1
	A4.Heggi.HEG.CEL	e1	c1
	A5.Heggi.CAW.CEL	c1	
	A6.Heggi.CEW.CEL	e3	c1
	A7.Heggi.HAW.CEL	e2	c1
	A8.Heggi.HEW.CEL	e1	c1
10	A1.jones.WT1.CEL	c1	
	A2.jones.WT2.CEL	c1	
	A3.jones.rh1.CEL	e1	c1
	A4.jones.rh2.CEL	e1	c1

11	A1.deeke.tum.CEL	e1	c1
	A2.deeke.Inf.CEL	c1	
	A3.deeke.tum.CEL	e1	c1
	A4.deeke.Inf.CEL	c1	
12	A1.MUT.Top1.CEL	e1	c1
	A2.MUT.Top2.CEL	e1	c1
	A3.MUT.Base1.CEL	e2	c2
	A4.MUT.Base2.CEL	e2	c2
	A5.Turner.WT.Top1.CEL	c1	
	A6.WT.Top2.CEL	c1	
	A7.WT.Base1.CEL	c2	
	A8.WT.Base2.CEL	c2	
13	A1.Fille.WT.nodex.CEL	c1	
	A2.Fille.WT..dex.CEL	e1	c1
	A3.Fille.ANGR4.12.CEL	e2	c1
	A4.Fille.ANGR4.12.dex.CEL	e3	c1
	A5.Fille.WTnodex.CEL	c1	
	A6.Fille.WT.dex.CEL	e1	c1
	A7.Fille.ANGR4.12nodex.CEL	e2	c1
	A8.Fille.ANGR4.12.dex.CEL	e3	c1
14	A10.Smith.17.CEL	c10	c9
	A11.Smith.21B.CEL	c1	c10
	A1.Smith.21A.CEL	c1	c10
	A2.Smith.22.CEL	c2	c1
	A3.Smith.23.CEL	c3	c2
	A4.Smith.1.CEL	c4	c3
	A5.Smith.5.CEL	c5	c4
	A6.Smith.8.45.CEL	c6	c5
	A7.Smith.10.CEL	c7	c6
	A8.Smith.11.CEL	c8	c7
	A9.Smith.13.CEL	c9	c8
	A10.smith.20h.CEL	c10	c9
	A11.smith.24h.CEL	c1	c10
	A1.smith.00h.CEL	c1	c10
	A2.smith.01h.CEL	c2	c1
	A3.smith.02h.CEL	c3	c2
	A4.smith.04h.CEL	c4	c3
	A5.smith.08h.CEL	c5	c4
	A6.smith.12h.CEL	c6	c5
	A7.smith.13h.CEL	c7	c6
	A8.smith.14h.CEL	c8	c7
	A9.smith.16h.CEL	c9	c8
15	A10.Bwoll.Col2.CEL	e4	c1
	A1.Bwoll.COG.CEL	c1	
	A2.Bwoll.C5G.CEL	e1	c1
	A3.Bwoll.COS.CEL	c1	
	A4.Bwoll.CSS.CEL	e1	c1

	A5.Bwoll.NG1.CEL	e2	c1
	A6.Bwoll.NG2.CEL	e2	c1
	A7.Bwoll.E11.CEL	e3	c1
	A8.Bwoll.E12.CEL	e3	c1
	A9.Bwoll.Col1.CEL	e4	c1
16	A1.WILLA.CON.CEL	c1	
	A2.WILLA.ISOX.CEL	e1	c1
	A1.willa.CON.REP2.CEL	c1	
	A1.willa.CON.REP3.CEL	c1	
	A2.willa.ISOX.REP2.CEL	e1	c1
	A2.willa.ISOX.REP3.CEL	e1	c1

Table: The first set of 153 microarrays that were used for this study are publicly available from the Nottingham Arabidopsis Stock Centre [60,61]. These belonged to 16 different series of experiments, that all included one or several experimental condition(s) (depicted as 'e') and at least one wildtype (controle, depicted as 'c').
