# Supplementary material for: Nonrandom divergence of gene expression following gene and genome duplications in the flowering plant Arabidopsis thaliana
Source: Genome Biol. 2006 Feb 20;7(2):R13. doi: 10.1186/gb-2006-7-2-r13 (PMC1431724; doi:10.1186/gb-2006-7-2-r13)
Supplement: Additional File 2 — This file contains the names of the microarrays that were included in the second dataset, together with the description of what tissue the samples were taken from and the conditions in which the plant was grown. [file gb-2006-7-2-r13-S2.pdf]

Dataset 2

Experiment	Tissue	Age	Photoperiod	Substrate
ATGE_1	cotyledons	7 days	continuous light	soil
ATGE_2	hypocotyl	7 days	continuous light	soil
ATGE_3	roots	7 days	continuous light	soil
ATGE_4	shoot apex, vegetative + young leaves	7 days	continuous light	soil
ATGE_5	leaves 1 + 2	7 days	continuous light	soil
ATGE_6	shoot apex, vegetative	7 days	continuous light	soil
ATGE_7	seedling, green parts	7 days	continuous light	soil
ATGE_8	shoot apex, transition (before bolting)	14 days	continuous light	soil
ATGE_9	roots	17 days	continuous light	soil
ATGE_10	rosette leaf #4, 1 cm long	10 days	continuous light	soil
ATGE_12	rosette leaf # 2	17 days	continuous light	soil
ATGE_13	rosette leaf # 4	17 days	continuous light	soil
ATGE_14	rosette leaf # 6	17 days	continuous light	soil
ATGE_15	rosette leaf # 8	17 days	continuous light	soil
ATGE_16	rosette leaf # 10	17 days	continuous light	soil
ATGE_17	rosette leaf # 12	17 days	continuous light	soil
ATGE_19	leaf 7, petiole	17 days	continuous light	soil
ATGE_20	leaf 7, proximal half	17 days	continuous light	soil
ATGE_21	leaf 7, distal half	17 days	continuous light	soil
ATGE_22	developmental drift, entire rosette after transition to flowering, but before bolting	21 days	continuous light	soil
ATGE_23	as above	22 days	continuous light	soil
ATGE_24	as above	23 days	continuous light	soil
ATGE_25	senescing leaves	35 days	continuous light	soil
ATGE_26	cauline leaves	21+ days	continuous light	soil

ATGE_27	stem, 2nd internode	21+ days	continuous light	soil
ATGE_28	1st node	21+ days	continuous light	soil
ATGE_29	shoot apex, inflorescence (after bolting)	21 days	continuous light	soil
ATGE_31	flowers stage 9	21+ days	continuous light	soil
ATGE_32	flowers stage 10/11	21+ days	continuous light	soil
ATGE_33	flowers stage 12	21+ days	continuous light	soil
ATGE_34	flowers stage 12, sepals	21+ days	continuous light	soil
ATGE_35	flowers stage 12, petals	21+ days	continuous light	soil
ATGE_36	flowers stage 12, stamens	21+ days	continuous light	soil
ATGE_37	flowers stage 12, carpels	21+ days	continuous light	soil
ATGE_39	flowers stage 15	21+ days	continuous light	soil
ATGE_40	flowers stage 15, pedicels	21+ days	continuous light	soil
ATGE_41	flowers stage 15, sepals	21+ days	continuous light	soil
ATGE_42	flowers stage 15, petals	21+ days	continuous light	soil
ATGE_43	flowers stage 15, stamen	21+ days	continuous light	soil
ATGE_45	flowers stage 15, carpels	21+ days	continuous light	soil
ATGE_73	mature pollen	6 wk	continuous light	soil
ATGE_76	siliques, w/ seeds stage 3; mid globular to early heart embryos	8 wk	long day (16/8)	soil
ATGE_77	siliques, w/ seeds stage 4; early to late heart embryos	8 wk	long day (16/8)	soil
ATGE_78	siliques, w/ seeds stage 5; late heart to mid torpedo embryos	8 wk	long day (16/8)	soil
ATGE_79	seeds, stage 6, w/o siliques; mid to late torpedo embryos	8 wk	long day (16/8)	soil
ATGE_81	seeds, stage 7, w/o siliques; late torpedo to early walking-stick embryos	8 wk	long day (16/8)	soil
ATGE_82	seeds, stage 8, w/o siliques; walking-stick to early curled cotyledons embryos	8 wk	long day (16/8)	soil
ATGE_83	seeds, stage 9, w/o siliques; curled cotyledons to early green cotyledons embryos	8 wk	long day (16/8)	soil
ATGE_84	seeds, stage 10, w/o siliques; green cotyledons embryos	8 wk	long day (16/8)	soil
ATGE_87	vegetative rosette	7 days	short day (10/14)	soil
ATGE_89	vegetative rosette	14 days	short day (10/14)	soil
ATGE_90	vegetative rosette	21 days	short day (10/14)	soil

ATGE_91	leaf	15 days	long day (16/8)	1x MS agar, 1% sucrose
ATGE_92	flower	28 days	long day (16/8)	soil
ATGE_93	root	15 days	long day (16/8)	1x MS agar, 1% sucrose
ATGE_94	root	8 days	continuous light	1x MS agar
ATGE_95	root	8 days	continuous light	1x MS agar, 1% sucrose
ATGE_96	seedling, green parts	8 days	continuous light	1x MS agar
ATGE_97	seedling, green parts	8 days	continuous light	1x MS agar, 1% sucrose
ATGE_98	root	21 days	continuous light	1x MS agar
ATGE_99	root	21 days	continuous light	1x MS agar, 1% sucrose
ATGE_100	seedling, green parts	21 days	continuous light	1x MS agar
ATGE_101	seedling, green parts	21 days	continuous light	1x MS agar, 1% sucrose

Table: The second set of microarrays that were used for this study contained data that were generated within the framework op the AtgenExpress project [39] and are publicly available from the Nottingham Arabidopsis Stock Centre [60,61]. The RNA was sampled from 63 different plant tissues in different stages.
