# Supplementary material for: Nonrandom divergence of gene expression following gene and genome duplications in the flowering plant Arabidopsis thaliana
Source: Genome Biol. 2006 Feb 20;7(2):R13. doi: 10.1186/gb-2006-7-2-r13 (PMC1431724; doi:10.1186/gb-2006-7-2-r13)
Supplement: Additional File 3 — This file contains the scatterplots of the Spearman correlation coefficient in function of the Ks value of all genes in the 67 different functional classes of genes. The loess smoother that was fitted to the data is depicted by a full black line, together with its 95% confidence interval. [file gb-2006-7-2-r13-S3.pdf]

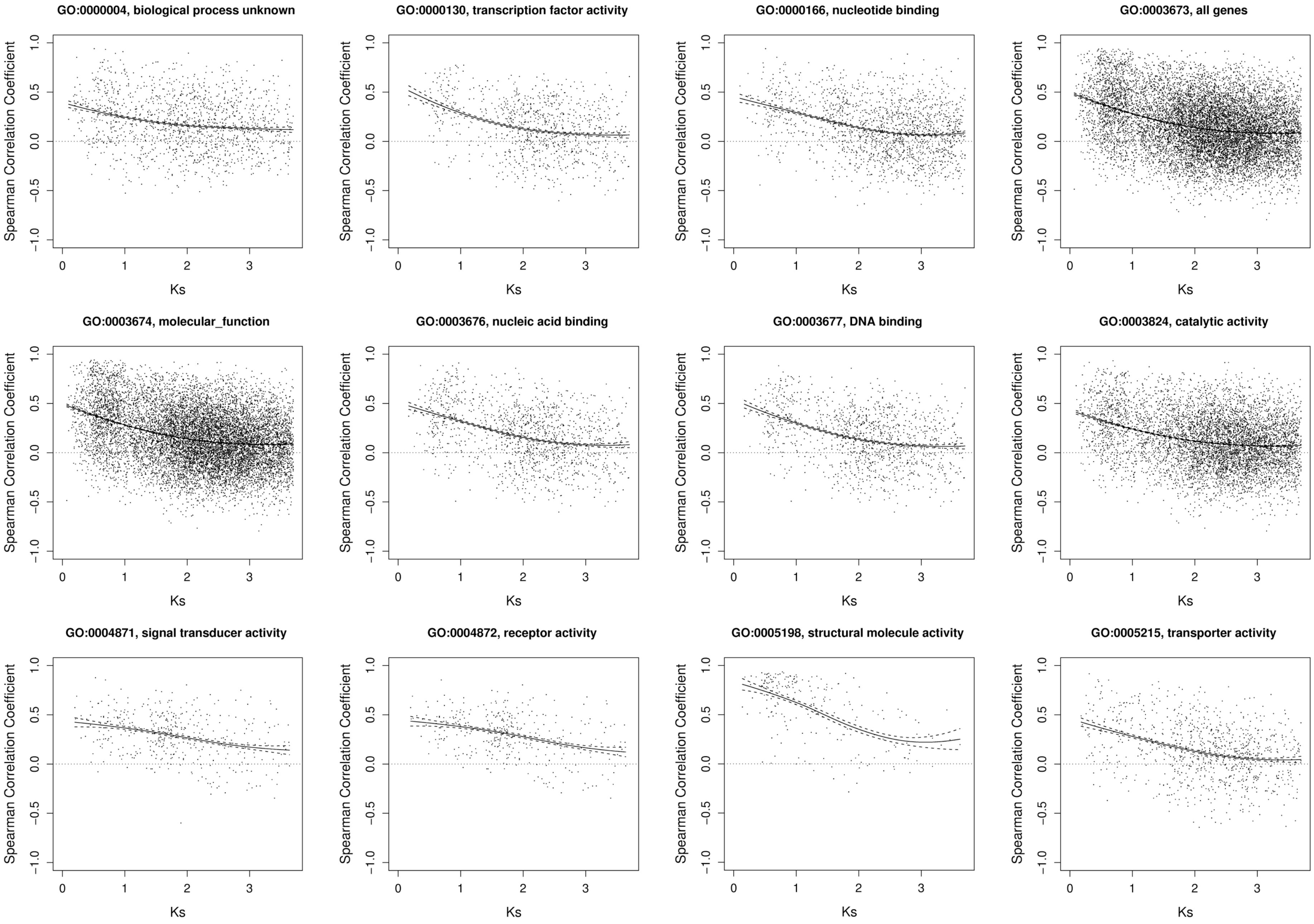

GO:0000004, biological process unknown
GO:0000130, transcription factor activity
GO:0000166, nucleotide binding
GO:0003673, all genes
GO:0003674, molecular_function
GO:0003676, nucleic acid binding
GO:0003677, DNA binding
GO:0003824, catalytic activity
GO:0004871, signal transducer activity
GO:0004872, receptor activity
GO:0005198, structural molecule activity
GO:0005215, transporter activity
Spearman Correlation Coefficient
Ks

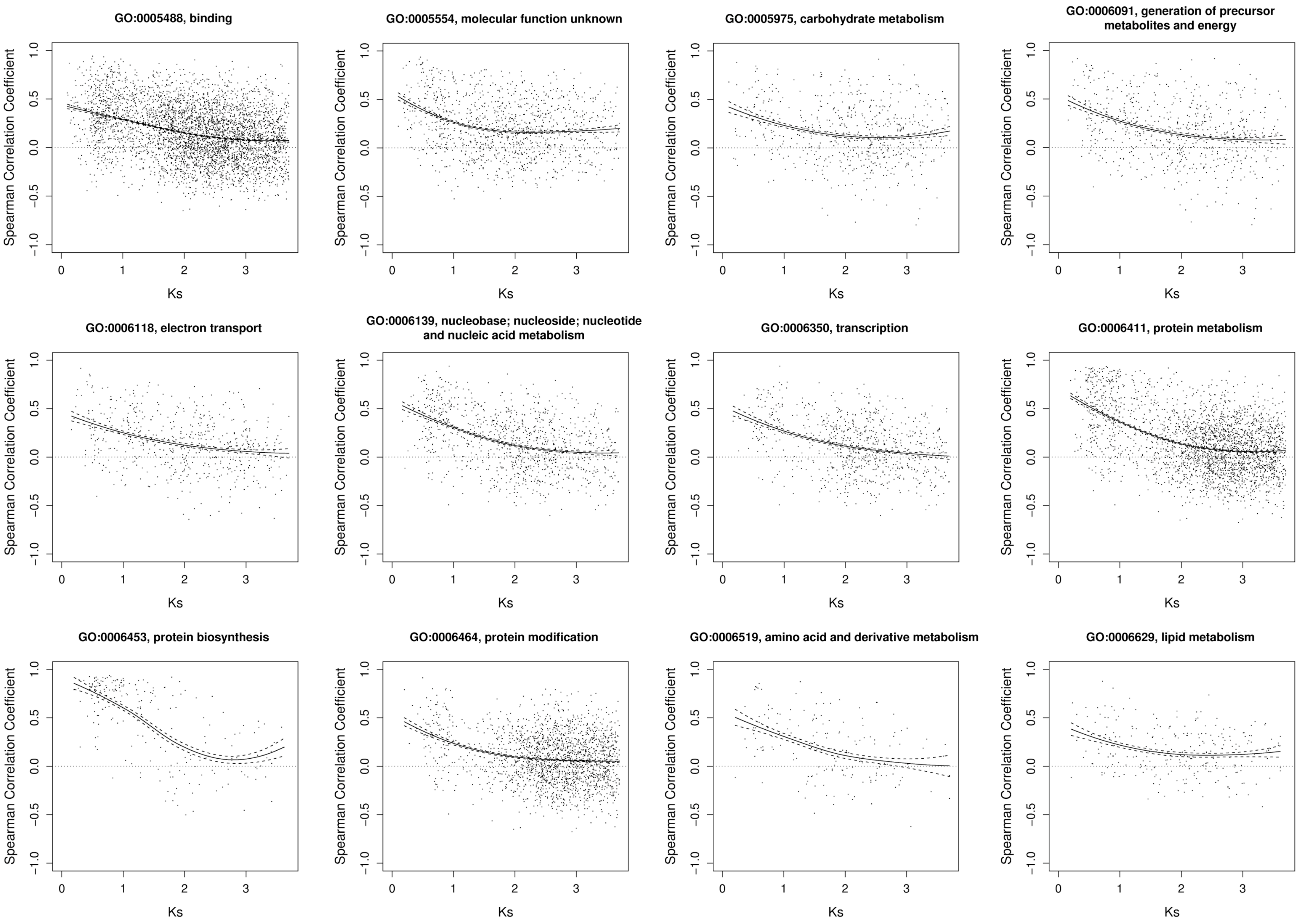
GO:0005488, binding
GO:0005554, molecular function unknown
GO:0005975, carbohydrate metabolism
GO:0006091, generation of precursor metabolites and energy
GO:0006118, electron transport
GO:0006139, nucleobase; nucleoside; nucleotide and nucleic acid metabolism
GO:0006350, transcription
GO:0006411, protein metabolism
GO:0006453, protein biosynthesis
GO:0006464, protein modification
GO:0006519, amino acid and derivative metabolism
GO:0006629, lipid metabolism
Spearman Correlation Coefficient
Ks

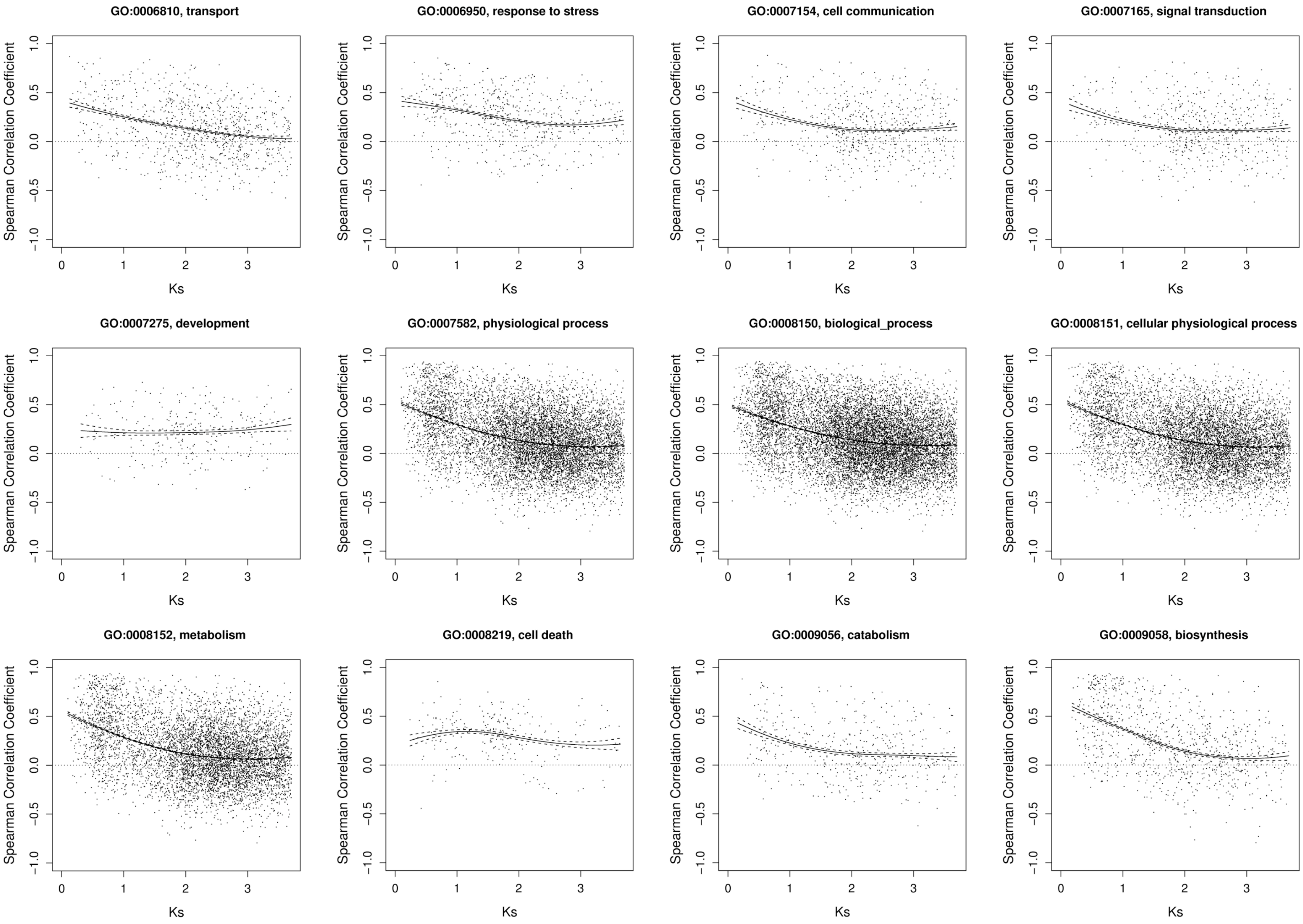
GO:0006810, transport
GO:0006950, response to stress
GO:0007154, cell communication
GO:0007165, signal transduction
GO:0007275, development
GO:0007582, physiological process
GO:0008150, biological_process
GO:0008151, cellular physiological process
GO:0008152, metabolism
GO:0008219, cell death
GO:0009056, catabolism
GO:0009058, biosynthesis
Spearman Correlation Coefficient
Ks

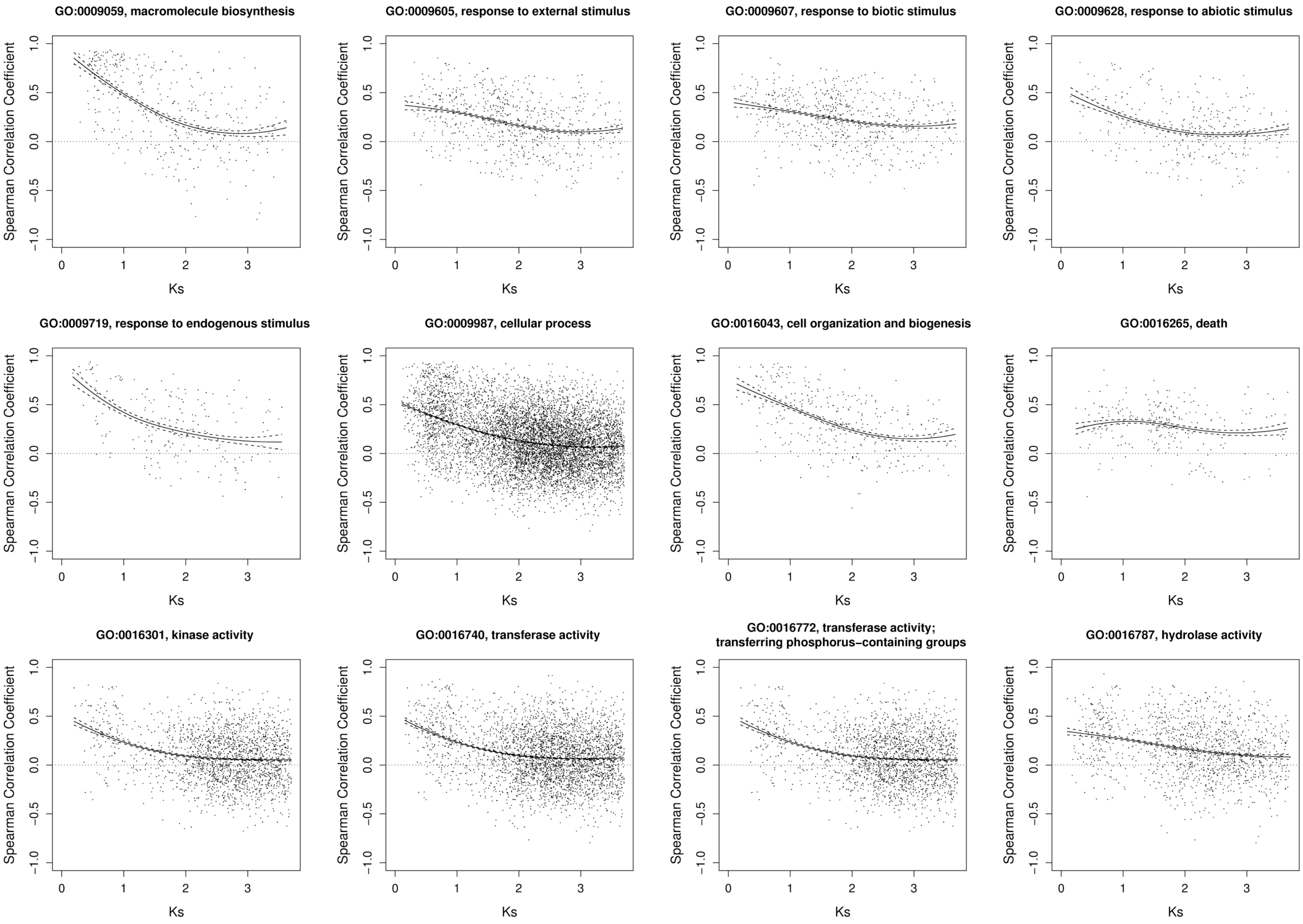
GO:0009059, macromolecule biosynthesis
GO:0009605, response to external stimulus
GO:0009607, response to biotic stimulus
GO:0009628, response to abiotic stimulus
GO:0009719, response to endogenous stimulus
GO:0009987, cellular process
GO:0016043, cell organization and biogenesis
GO:0016265, death
GO:0016301, kinase activity
GO:0016740, transferase activity
GO:0016772, transferase activity; transferring phosphorus−containing groups
GO:0016787, hydrolase activity
Spearman Correlation Coefficient
Ks
−1.0
−0.5
0.0
0.5
1.0
0
1
2
3

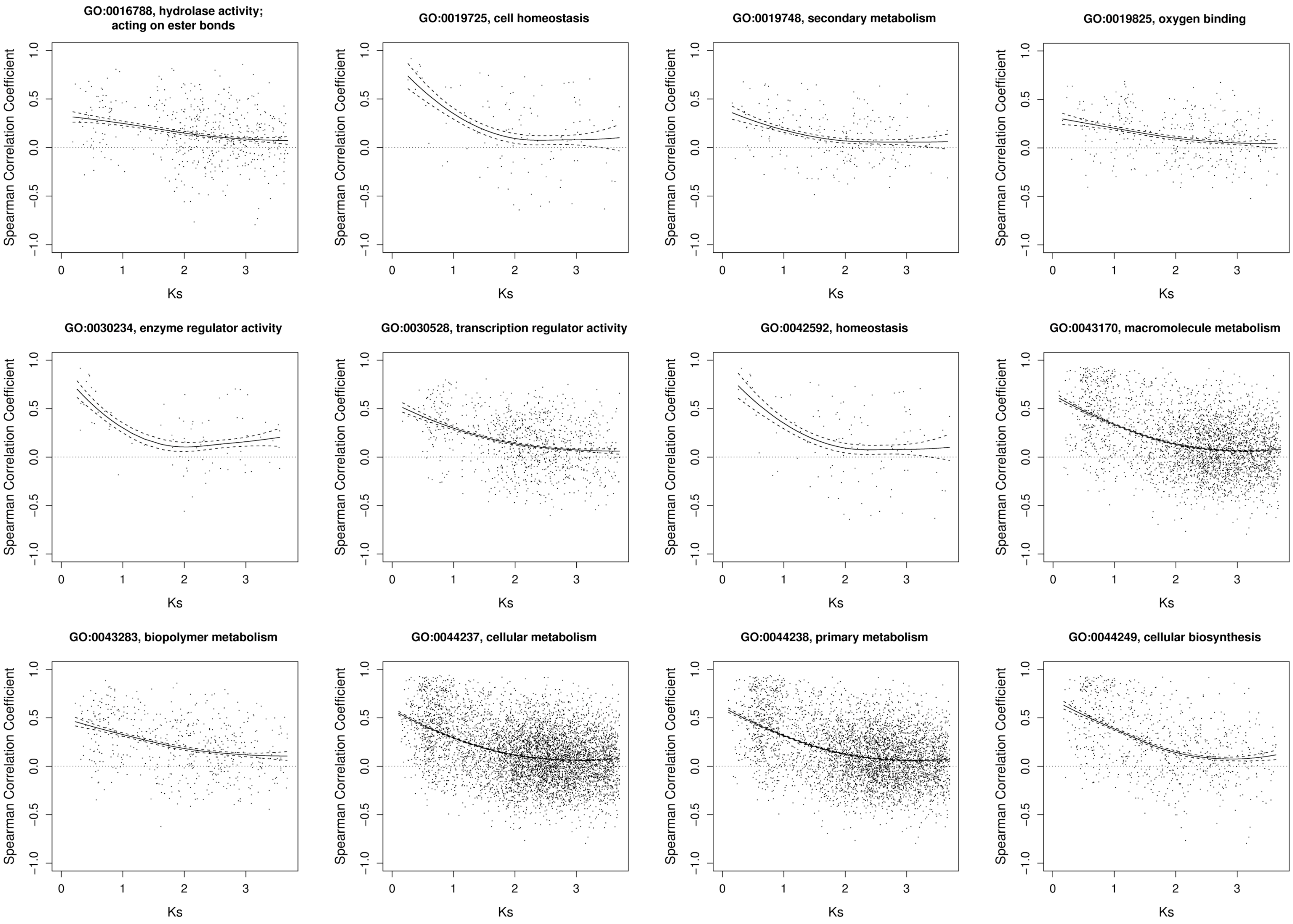
GO:0016788, hydrolase activity;
acting on ester bonds
GO:0019725, cell homeostasis
GO:0019748, secondary metabolism
GO:0019825, oxygen binding
GO:0030234, enzyme regulator activity
GO:0030528, transcription regulator activity
GO:0042592, homeostasis
GO:0043170, macromolecule metabolism
GO:0043283, biopolymer metabolism
GO:0044237, cellular metabolism
GO:0044238, primary metabolism
GO:0044249, cellular biosynthesis
Spearman Correlation Coefficient
Ks

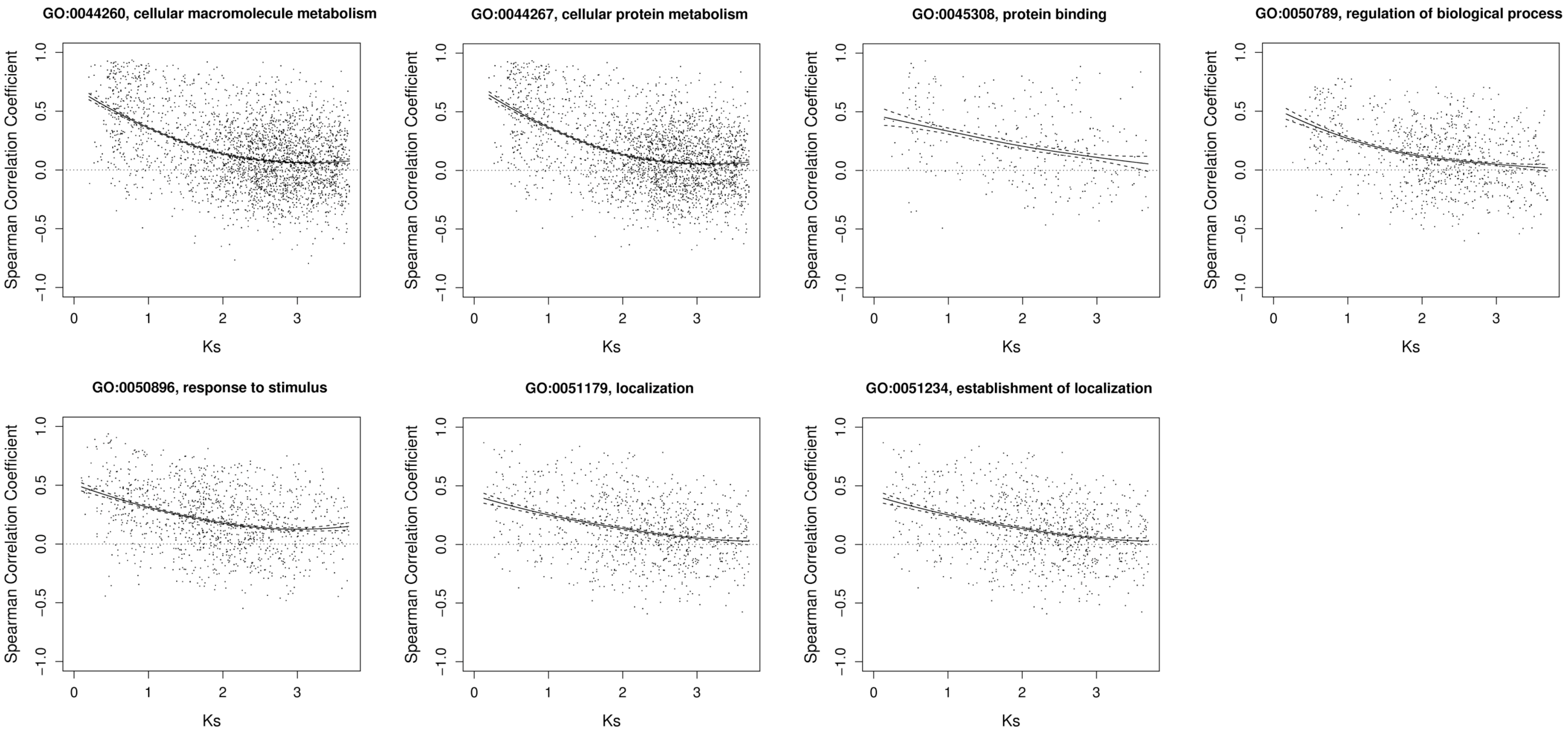
GO:0044260, cellular macromolecule metabolism
GO:0044267, cellular protein metabolism
GO:0045308, protein binding
GO:0050789, regulation of biological process
GO:0050896, response to stimulus
GO:0051179, localization
GO:0051234, establishment of localization
Spearman Correlation Coefficient
Ks
1.0
0.5
0.0
−0.5
−1.0
0
1
2
3
